# Preparation of Anti-Tumor Nanoparticle and Its Inhibition to Peritoneal Dissemination of Colon Cancer

**DOI:** 10.1371/journal.pone.0098455

**Published:** 2014-06-04

**Authors:** Qingchao Tang, Yihui Wang, Rui Huang, Qi You, Guiyu Wang, Yinggang Chen, Zheng Jiang, Zheng Liu, Lei Yu, Shan Muhammad, Xishan Wang

**Affiliations:** 1 Department of Colorectal Cancer Surgery, Cancer Center, The Second Affiliated Hospital, Harbin Medical University, Harbin, China; 2 Department of Colorectal Surgery, The Third Affiliated Hospital, Harbin Medical University, Harbin, China; RMIT University, Australia

## Abstract

**Background:**

5-Fluorouracil (5-FU) is one of the most classic chemotherapy drugs. Nanoparticle drug delivery vehicles offer superiority over target effect enhancement and abatement of side effects. Little is known however as to the specific effect of nanoparticle on peritoneal dissemination of colon cancer. The aim of this study is to prepare one NPs (nanoparticles) loaded with 5-FU and investigate the characteristic of NPs and the role of it in peritoneal metastasis nodules formation of human colon cancer.

**Methodology/Principal Findings:**

Prepared the NPs (nanoparticles) loaded with 5-FU (5-Fluorouracil) by PEG-PLGA with the method of double emulsion. Then evaluate the characteristics of the NPs by scanning electron microscopy, analyzing the particle diameter distribution and determining the loading efficiency. Detect the release features of NPs in vitro and in vivo. Nude mice with peritoneal metastases were treated with 5-FU solution or 5-FU-NPs through peritoneal cavity. Count the nodules on peritoneum and mesenterium and survey the size of them. We got NPs with average-diameter of 310 nm. In vitro release test shows NPs can release equably for 5 days with release rate of 99.2%. In vivo, NPs group can keep higher plasma concentration of 5-FU longer than it in solution group. The number of peritoneal dissemination nodule below 1 mm in 5-FU-sol group(17.3±3.5) and 5-FU-NP group(15.2±3.2) is less than control group(27.2±4.7)(P<0.05). The total number of nodules in 5-FU-NP group(28.7±4.2) is significantly smaller than in 5-FU-sol group(37.7±6.3) (P<0.05).

**Conclusions/Significance:**

The novel anti-tumor nanoparticles loaded with 5-FU by PEG-PLGA can release maintain 5 days and have inhibitory action to peritoneal dissemination of colon cancer in mice.

## Introduction

Colorectal cancer is the third leading cause of cancer-related deaths worldwide. Peritoneal metastasis of colorectal cancer is common with incidence of about 13% which were reported in study on large sample previously [1].Peritoneal metastasis occurred in 7% of patients with colorectal cancer in the initial treatment and in 4%∼19% patients after radical surgery [2].The prognosis of colorectal cancer peritoneal metastasis is poor whose median survival is only 5∼9 months [3].The current systemic chemotherapy regimens based on 5-FU for colorectal cancer have not achieved satisfactory results, particularly in the treatment of peritoneal dissemination [4]. One of the problems with this type of therapy is the limited delivery of systemically administered drugs to the peritoneal [5]. Direct intraperitoneal administration may cause 5-FU absorbed into blood circulation rapidly result in the insufficient dose arrive at local nodule in peritoneal cavity. It is necessary to develop new strategies for the treatment of peritoneal dissemination in colorectal cancer to achieve better results. Nanoparticle, as a novel carrier for anti-tumor drugs, has been paid a close attention to by the medical field in early 1978 till now [6]. In recent years, the studies on polymer nanoparticle have made a tremendous advancement In virtue of the biocompatibility and biodegradability of polymer nanoparticles [7]. The polymeric spheres can protect the drug from adverse external conditions and control its release [8]. Compared with microspheres, NPs have their own superiority over target effect enhancement and abatement of side effects [9]–[10]. Neovessels in tumor are more permeable for nanoparticles under 400–600 nm to pass, which not only can improve the target function but also can lessen the side effects of anti-tumor drugs[11]. While,the intraperitoneal administration of nanoparticle antitumor agents for the treatment of colorectal cancer peritoneal dissemination has not been investigated extensively. In view of this, we prepared the 5-FU nanoparticles with a novel technique firstly, and demonstrated anti-tumor nanoparticles can inhibit formation of peritoneal dissemination of colorectal cancer.

## Materials and Methods

### Ethics Statement

All animal experiments were approved by the Institutional Animal Care and Use Committee and Ethics Committee of Harbin Medical University and in accordance with the guidelines of the Animal Experiment Center of Harbin Medical University.

### Preparation of 5-FU/PEG-PLGA nanoparticles

To begin with, PLGA-PEG is added into 80 ml dichloromethane. 4 ml of 10% (w/w) NaOH solution containing 5-FU is slowly injected into mixture under high shearing emulsification (Fa25 emulsifier, Fluko, USA), slightly transparent emulsion was thus obtained. Dripped them into 160 ml of 5-FU saturated solution containing 5% (w/v) of PVA under a vigorous stirring (Fa25 emulsifier, Fluko, USA) for 5 mins in order to obtain the double emulsion (w/o/w). The solvent evaporation was carried out under vacuum with a rotating evaporator (RE-85A rotating evaporator, Henan Yuxin Instrument corporation). NPs are recovered by means of centrifugation at 12000 rpm, and later washed with 5-FU saturated aqueous solution and distilled water which were all lyophilized at last.

### Evaluate quality of NPs

#### Evaluate the morphology characteristic of NPs

1 mg NPs are dispersed into 1 ml water. NPs suspensions were dropped on the slide and spurted gold on when it is dry. The morphology of the NPs is investigated by scanning electron microscopy (JSM-6700F, JEOL, and Japan).

#### Analysis of particle Size

Particle size distribution is determined by laser size analysator (LS-13320 laser size analysator, BeckmanCoulter, USA). Each product is analyzed for 30 times after suspended in distilled water.

#### Determination of Encapsulating Efficiency

5-FU loading efficiency is determined by thermo gravimetric analysis (STA409 thermal analyzer, Germany). A certain amount of dry NPs was heated at the heating rate of 10°C/min under nitrogen atmosphere.

### Evaluating the in vitro Release Character of NPs

50 mg of 5-FU NPs was dispersed in 10 mL of PBS(pH = 7.4). This solution is added into a dialysis bag, which was put into 90 mL of PBS(pH = 7.4), sealed, and agitated(∼75 r/min) at 37 °C. A sample of 10 mL was collected at specified time intervals from outer PBS and supplement equal amounts of fresh PBS.

### Evaluating the In Vivo Release Character of NPs by HPLC

Kunming mice were purchased from the Shanghai Laboratory Animal Center (Shanghai, China). Mice that were housed under identical conditions were allowed free access to a standard diet and tap water and exposed to with a 12-h light: 12-h dark cycle. 90 Kunming mice are divided into 2 groups at random. 5-FU-NPs group (n = 45) is the experiment group, while the contrast group is 5-FU solution group, both of which are divided respectively into 9 teams with 5 mice by randomized method. Treat the mice by way of intra-abdominal with the dose of 40 mg/kg. To alleviate suffering, mice were performed with inhalation anesthesia by ethylether before getting blood. The blood and put into heparinized centrifuge tube for 5 min at 3000 rpm from eye sockets correspondingly at 0.083,0.167,0.333,0.5,1.0,1.5,2.0,3.0,4.0 h. Take the supernatant liquid 200 µl into EP tube into with 25 µl internal control (50 µg/ml 5-Bru solution) and 800 µl acetic ether precisely were added precisely,mixed uniformity under turbulent agitation for 1 min, and centrifuged for 5 min(5000 rpm). Filter the supernatant liquid by micropore film (0. 45 µm), and then dry it by nitrogen gas and dissolve it by mobile phase 100 µl. Ultimately, the sample is injected into HPLC, so that we can calculate the plasma concentration of 5-FU.

### Cell culture

The human colon cancer cells HCT116 purchase from “The cell resources center of Shanghai Institute of life science, Chinese Academy of Sciences”,which were maintained in 1640 medium that was supplemented with 10% fetal bovine serum, 100 U/ml penicillin G and 100 lg/ml streptomycin at 37°C in a humidified incubator containing 5% CO2.

### Cell viability assay

Cell viability was determined using a 3-(4,5-dimethylthiazole-2-yl)-2.5-diphe-145 nyltetrazolium bromide (MTT) assay as described previously [12].

### Colony formation assay

HCT116 cells were seeded in 3.5-cm dishes (1000 cells/dish) and cultured for 2 weeks to allow for colony formation. 5-FU and 5-FU-NPs were added in dishes separately. The colonies were fixed in methanol, stained with 0.1% crystal violet and counted.

### Evaluation of 5-FU-NPs Effects on peritoneal dissemination in mouse model

BALB/c-nu nude mice (aged 4 weeks) were purchased from the Shanghai Laboratory Animal Center (Shanghai, China). All animal experiments were approved by the Institutional Animal Care and Use Committee of the Harbin Medical University. Nude mice were housed in SPF under identical conditions and allowed free access to a standard diet and tap water and exposed to with a 12-h light: 12-h dark cycle. HCT116 cells(5×10^5^/mouse)/ml saline were injected into the peritoneal cavity of 4-week-old(BALBc nu/nu) mice. Five mice were allocated to each group. 5-FU-sol and 5-FU-NPs were administered in peritoneal cavity at MTD of 40 mg/kg body weight weekly from day 7. Mice were sacrificed on days 28 after anesthetized with ethyletherfor alleviate suffering. The number of peritoneal nodules was counted respectively according to diameter under 1.0 mm or over 3.0 mm by microscopy and data are shown from representative experiments.Tumor volume was calculated based on the formula V  = π×L×S×S/6 (L, the long axis; S, the short axis).

### Cell cycle and apoptosis analysis with flow cytometry

See supplemental Methods in File S1.

## Results

### Analyze the morphology, particle size distribution, and encapsulating efficiency of 5-FU-NP

**Figure 1:**
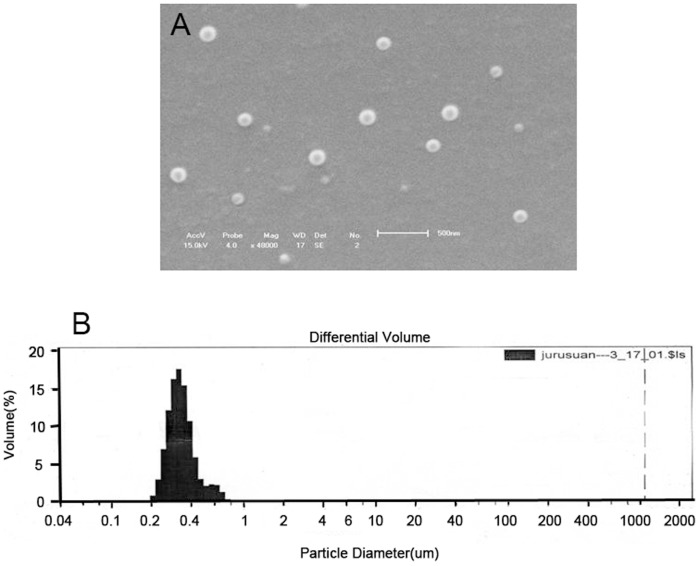
Analyze the morphology of 5-FU-NPs. (a) SEM scan showed the NPs with spherical shape and smooth surface. (b) Nanoparticle size was analyzed by laser size analysator. Results showed average-diameter of NPs is about 310 nm. Distribution of nanoparticles was range from 255 nm to 469 nm and 70% were less than 385 nm. P<0.05.

As the SEM photo presents (Figure 1A) the morphology of NPs reveals a spherical or elliptic structure with smooth surface and not adherent with each other. SEM photo of NPs in 10000 and 48000 amplification was shown in Figure.S1 in File S1. Particle size is demonstrated in particle size determination, with the average-diameter of 310 nm is well-distributed (Figure 1B). The original parameters of laser-size analysis for NPs were listed in Figure.S2 in File S1. Encapsulating efficiency of NPs is(15.38±0.56)% detected from 5 samples.

### In vitro Release of 5-FU from NPs

**Figure 2:**
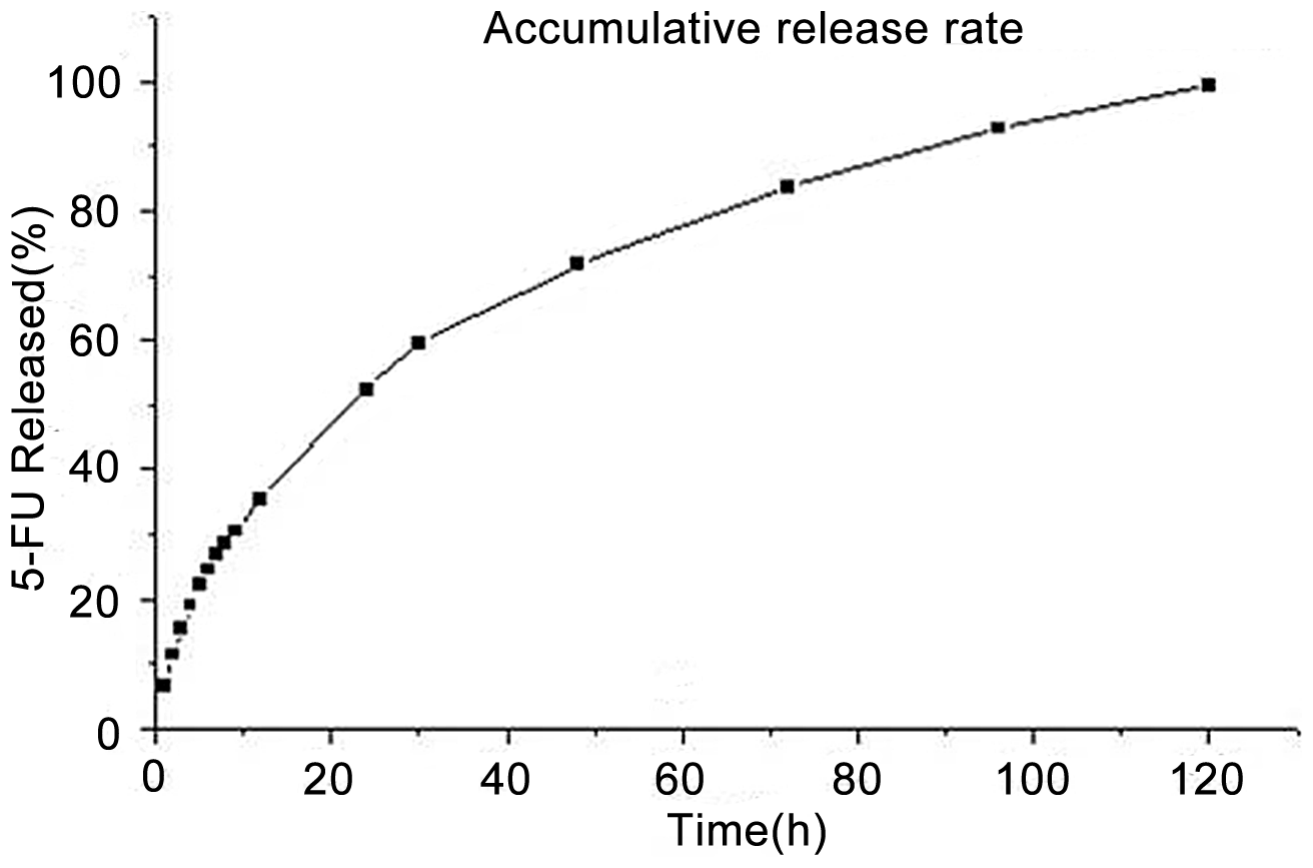
In vitro pharmacokinetics test were performed in PBS which showed that 5-FU-NPs may release maintain for about 5 days evenly with accumulating release amount up to 99.2%. P<0.05.

The result of in vitro release of 5-FU from NPs is shown in (Figure.2). 5-FU can release maintain for 5 days with accumulating release amount up to 99.2%.Linear fit of Q to release time(t) gets the release function: Q = 20.9037+0.80953*t.

### 5-FU-NP Pharmacokinetics in vivo

**Figure 3:**
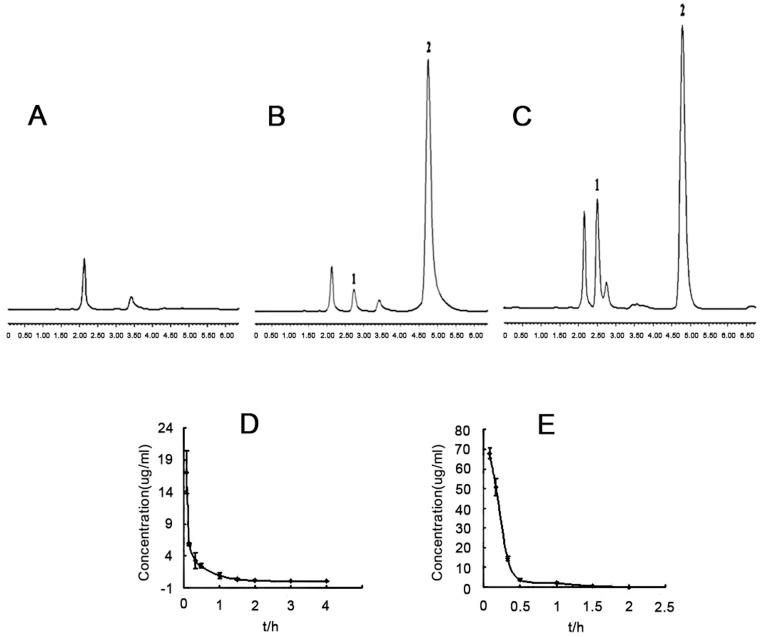
In vivo pharmacokinetics test were performed in mice by high-performance liquid chromatography. Chromatogram was shown in (a) for blank plasma, (b) for plasma with 5-FU and 5-Bru, and (c) for sample in plasma with 5-FU and 5-Bru.(5-Bru as endo-parameter). The C-T-curve indicated that the mean residence time for (d) 5-FU-NPs is 0.490 longer than (e) 5-FU solution (0.271 h). And the concentration can not be identified 2 h later for 5-FU solution group.

In this research, the retention time of 5-FU is 4.6 min which is illustrated in chromatogram (Figure 3A,3B,3C). Standard curve equations of Y(peak area ratio of 5-FU and internal standard)and C is Y = 0.06266C+0.01752(R = 0.99687)(n = 5), the lowest detectable limit is 0.05 µg/ml, precision RSD at low, medial and high concentration is 9.05%,4.73%,2.97% respectively,and recovery rate is 87.43%,102.91%,108.64% respectively.

Figure.3 illustrates the flow of C-T in plasma after the mice have been administrated in abdomen (Figure 3D,3E). The area of C-T curve(AUC0∼t) when the mice are administrated and the mean residence time(MRT0∼t)are calculated by the use of pharmacokinetics statistical software DAS2.0, and then carry on the T-test with SPSS10.0 software (Table 1).

**Table 1:** Parameters after the administration of 5-Fu and 5-FU-NPS in plasma of mice.

Parameters	5-FU-NP(n = 5)	5-FU solution(n = 5)
T1/2/h	0.237±0.023*	0.716±0.181
*AUC*_0∼t_/mg·h·L^−1^	4.372±0.546*	17.147±1.076
*AUC*_0∼∞_/mg·h·L^−1^	4.405±0.540*	17.188±1.059
*MRT*_0∼t_/h	0.490±0.023*	0.271±0.007
*MRT*_0∼∞_/h	0.527±0.044*	0.276±0.006
*C*_max_/mg/L	17.063±3.327*	67.769±2.678

5-FU-NP compared with 5-FU solution by T-test:*P<0.01.

### 5-FU-NPs suppresses the growth of colorectal cancer cells

**Figure 4:**
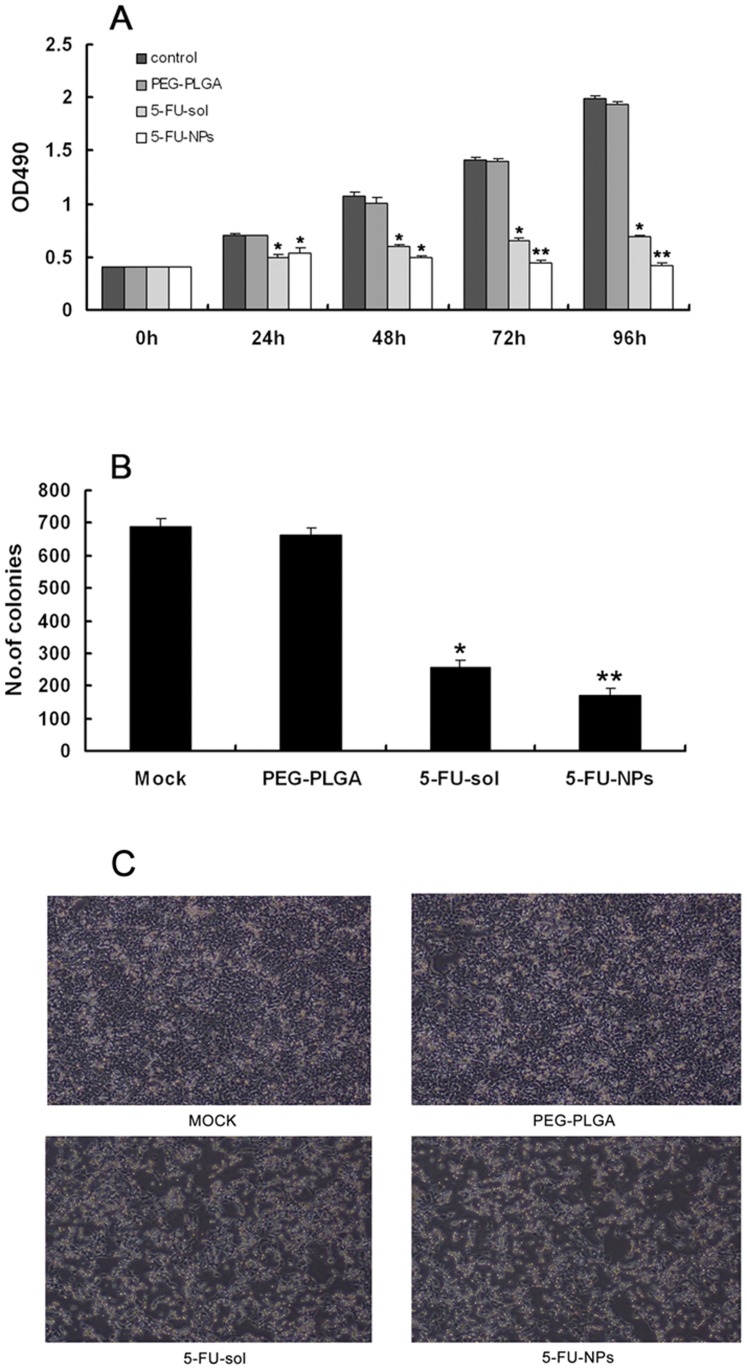
Anti-tumor nanoparticles inhibit the proliferation of colon cancer cells. (a) HCT116 cell line were added with 5-FU-NP or 5-FU-sol or PEG-PLGA or mock and subjected to a cell-viability assay in 24 h, 48 h, 72 h and 96 h. (b) A clone formation assay was performed in mock, PEG-PLGA, 5-FU-sol and 5-FU-NPs groups. * P<0.05,versus control. ** P<0.05, versus 5-FU-sol. (c) Representative photos after adding with PEG-PLGA, 5-FU-sol and 5-FU-NPs and mock.

To investigate how 5-FU-NPs affect the proliferation of colon cancer cells, we performed MTT and clone formation assays in HCT116 cells. MTT results showed that the number of viable cells in 5-FU-NPs were significantly fewer than that of negative control cells at 24 h,48 h, 72 h, 96 h which also fewer than that of 5-FU-sol group at 48 h, 72 h, 96 h (Figure 4A). As expected, 5-FU-NPs inhibited the growth rate of cells compared to control group. Cells then were cultured for 14 days to perform clone formation assay. As the results showed that 5-FU-sol and 5-FU-NPs both can decrease colony numbers compared to control group. Farther more, 5-FU-NPs resulted in a greater than 35% decrease in colony numbers compared to the 5-FU-sol group (Figure 4B). Cell cycle were blocked by both 5-FU solution and 5-FU-NPs compared with control group (Figure S3 in File S1). Furthermore, apoptosis analysis assay were also performed to declare how 5-FU-NPs effected on ability of colon cancer cell. (Figure S4 in File S1) The flow cytometry results showed that 5-FU-sol and 5-FU-NPs might promote apoptosis of colon cancer cells compare with mock or PEG-PLGA. As the results showed that 5-FU-NPs might enhance the effects of 5-FU on cell cycle or apoptosis partly compared with 5-FU-sol, but the effects were not significantly.

### Inhibitory effect of 5-FU-NP on peritoneal dissemination formation of colorectal cancer in vivo

**Figure 5:**
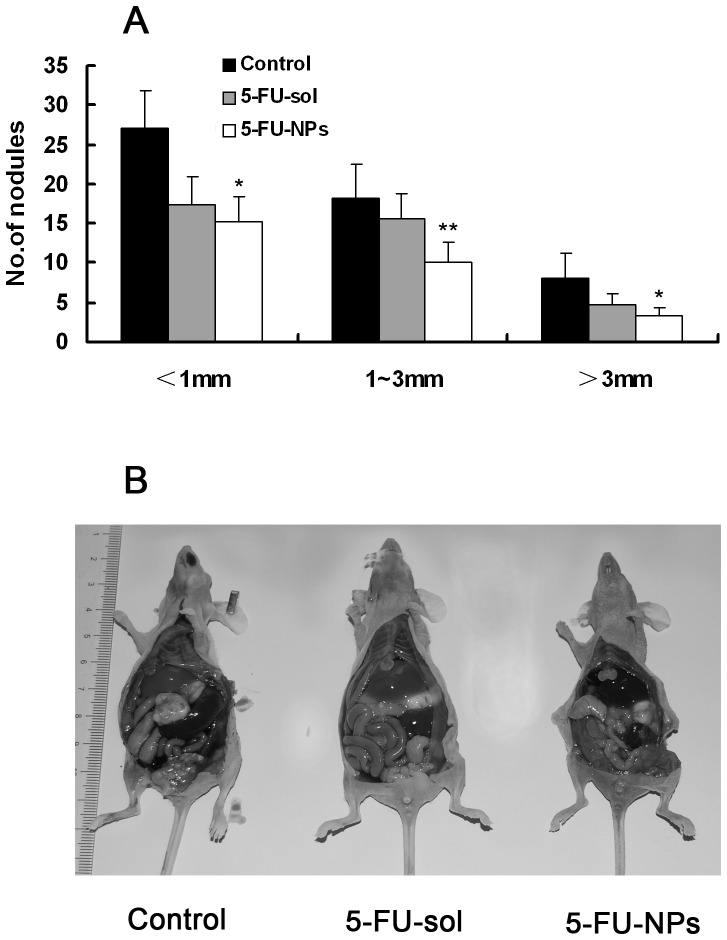
Anti-tumor nanoparticles may inhibit nodules formation of colon cancer peritoneal metastasis in nude mice. (a) Metastatic nodules in abdominal cavity were counted according to diameter less than 1 mm, 1∼3 mm or more than 3 mm. (b) Visible tumor nodules were present in the abdominal cavity in representative photos of nude mice were sacrificed 4 weeks after i.p. injection with HCT116 cells.

The effect of 5-FU-NPs on peritoneal dissemination was evaluated. Macroscopic dessemination with visible tumor nodules were present in the abdominal cavity (Figure.5B). All of the mice were sacrificed on day 14, and the number of tumor nodules in the mesentery were counted (Table.2). The mean number of metastatic nodules less than 1 mm in 5-FU-sol group(17.4±3.6) and 5-FU-NP group(15.2±3.2) is smaller comparing with control group(27.2±9.7)(P<0.05). The total number of nodules in 5-FU-NP group (28.7±5.5) is significantly smaller than in 5-FU-sol group(37.6±5.4) (P<0.05).The incidences of peritoneal dissemination were 100% in all groups. The incidences of liver metastasis in 5-FU-NP group (33.3%) is lower than control group (100%) (p<0.05) (Figure.5A).

**Table 2:** Antitumour effect of 5-FU-NP on mice peritoneal metastasis.

	Incidence of Metastasis(%)	Number of nodules on mesenterium			
		Nodule diameter(mm)			Total
		<1	1–3	>3	
Control (n = 6)	100	27.2±4.7	18.2±4.3	8.2±3.2	53.5±9.4
5-Fu-sol (n = 6)	100	17.3±3.5*	15.7±3.1	4.7±1.5	37.7±6.3*
5-Fu-NP (n = 6)	100	15.2±3.2*	10.2±2.4**	3.3±1.1*	28.7±4.2**

Compared with control group,*p<0.05,Compared with sol group,**p<0.05.

## Discussion

Peritoneal dissemination is serious problem for advanced colon cancer patient because of its poor prognosis and the lack of effective treatment [13]. 5-FU, one of the most classic anti-tumor drugs,which can restrain the DNA synthesis processing of tumor cell, is widely used in digestive system cancer [14]–[15]. However, dose-dependent toxicity limited the common administration by venous injection and less dosage arriving at peritoneal nodules through systemic circulation result in poor treatment effect for peritoneal metastasis of colon cancer [16]–[17]. Therefore, it is imperative to find the novel strategy for peritoneal metastasis of colon cancer. In the present study, we prepared one anti-tumor NPs load with 5-FU may contribute to the cure of peritoneal dissemination of colon cancer.

The 5-FU-NPs were prepared by high shearing emulsification instead of the ultrasonic emulsification, which enlarged emulsifying energy so that obtained NPs with diameter of 310 nm (Figure1A). Our previous studies showed that we can obtain NPs in different size by adjusting internal water phase volume and dispersion time [18]. Compared with using ultrasonic emulsification reported before, it simplifies the process of the experiment and overcomes the restriction of the relative low output because of the exertion of the ultrasonic emulsification [19]. Furthermore, the particle size of microsphere is effectively reduced and well distributed. Examined from the particle size distribution, the diameter of 70% nanoparticle is less than 385 nm with the distribution range from 255 nm to 469 nm (Figure1B). We attempts to replace PLA with PLGA (50∶50) and the chain of PEG from 5000 to 2000, which contribute to create well-distributed 5-FU-NPs whose encapsulating efficiency was 15.4%. In respect that PEG-PLGA with favorable histocompatibility and biodegradability, they are generally employed in the medical and biological carriers [20]–[21]. This research illustrates that this 5-FU-NPs can keep releasing maintain for about 5 days slowly and smoothly in vitro which simulated human body environment inside the abdominal cavity(Figure 2).

To declare the in vivo releasing character of this novel 5-FU-NPs, pharmacokinetics experiments were practiced in vivo. The drugs encapsulated by NPs, a new type of medical carrier, are usually taken in by liver and spleen firstly,which contains abundant endothelial system [22]–[23]. According to the study in mice, we found that there exists a kind of disharmonious compartment model between different individual data and blood drug level. 5-FU-NP shows two compartment model in vivo comparing with the control group presenting one compartment model, which results in the application of statistical moment. The mean residence time(MRT(0-t))of 5-FU-NP group is 0.490 h (P<0.01), which is obviously longer than the control group (0.271 h)(Figure 3D,3E). It is shown that 5-FU concentration can not be identified in 2 h for control group, which demonstrates common 5-FU was decomposed in vivo shortly. The area in C-T curve (AUC (0-t)) is 4.372 mg/L•h Cmax is 17.06 µg/ml in 5-FU-NPs group compared with the control group is 67.77 µg/ml, which may result from precedence intake in target tissue for drugs loaded by nanoparticles(Table 1).

Anti-tumor NPs may inhibit tumor metastasis in abdominal cavity has been indicated in many studies [24]–[25]. However, it is not clear how the treatment effect of anti-tumor nanoparticles for colon cancer peritoneal dissemination. The MTT studies confirmed our hypothesis that 5-FU nanoparticle therapeutics are more effective than normal 5-FU solution especially after 48 h (Figure4A). The same results were showed in clone formation assay (Figure 4B).On the other hand, the function of 5-FU effect on apoptosis or cell cycle were neither enhanced by 5-FU-NPs compared with 5-FU-NPs, which were proved in apoptosis and cell cycle analysis by flow cytometry methods (Figure. S3, S4 in File S1). As shown in Figure 4C, 5-FU-sol and 5-FU-NPs led to altered morphological characteristics compare with mock, which identified by a scattered distribution of cells in the culture and a spindle- or star-like morphology of the cells (Figure4C). This change of morphological characteristics may be result from 5-FU-NPs effect on epithelial-mesenchymal transition function in cancer cells [26], which should be study in the future. On the other hand, to demonstrate in vivo efficacy of 5-FU-NPs therapeutics for peritoneal dissemination, we developed a peritoneal metastasis model to mimic the human condition. The numbers of nodules were evaluated by diameter and divided into 3 groups. The results illustrates that 5-FU-NPs have the ability to inhibit nodule formation superior to both positive control group and negative control group (Figure 5A,5B).

In conclusion, to achieve a better prognosis for patients with intraperitoneal metastases, the establishment of efficient treatment strategy is crucial. In this study, we successfully prepared anti-tumor nanoparticles loaded with 5-FU, which demonstrated inhibition effects in the colon cancer cell line and peritoneal disseminations in nude mouse model. Therefore, it may be a novel anti-tumor preparation for peritoneal metastases with profound prospects in clinical application. Still it has to be further researched for the detail mechanism in future.

## Supporting Information

File S1 **1. Supplementary results.** Fig.S1 SEM scans of 5-FU-NPs. Fig.S2 5-FU NPs size analysis. Fig.S3 Cell cycle blocked by 5-FU-NPs. Fig.S4 Apoptosis promoted by 5-FU-NPs. **2. Supplementary methods.** Laser size analysis of Particle. Flow cytometry and cell cycle analysis. Flow Cytometry apoptosis analysis with PI and annexin V staining
(DOC)
