# Supplementary material for: Preparation of Anti-Tumor Nanoparticle and Its Inhibition to Peritoneal Dissemination of Colon Cancer
Source: PLoS One. 2014 Jun 4;9(6):e98455. doi: 10.1371/journal.pone.0098455 (PMC4045714; doi:10.1371/journal.pone.0098455)
Supplement: File S1 — 1. Supplementary results. Fig.S1 SEM scans of 5-FU-NPs. Fig.S2 5-FU NPs size analysis. Fig.S3 Cell cycle blocked by 5-FU-NPs. Fig.S4 Apoptosis promoted by 5-FU-NPs. 2. Supplementary methods. Laser size analysis of Particle. Flow cytometry and cell cycle analysis. Flow Cytometry apoptosis analysis with PI and annexin V staining (DOC) [file pone.0098455.s001.doc]

1. **Supplementary results**

**Fig.S1 SEM scans of 5-FU-NPs**


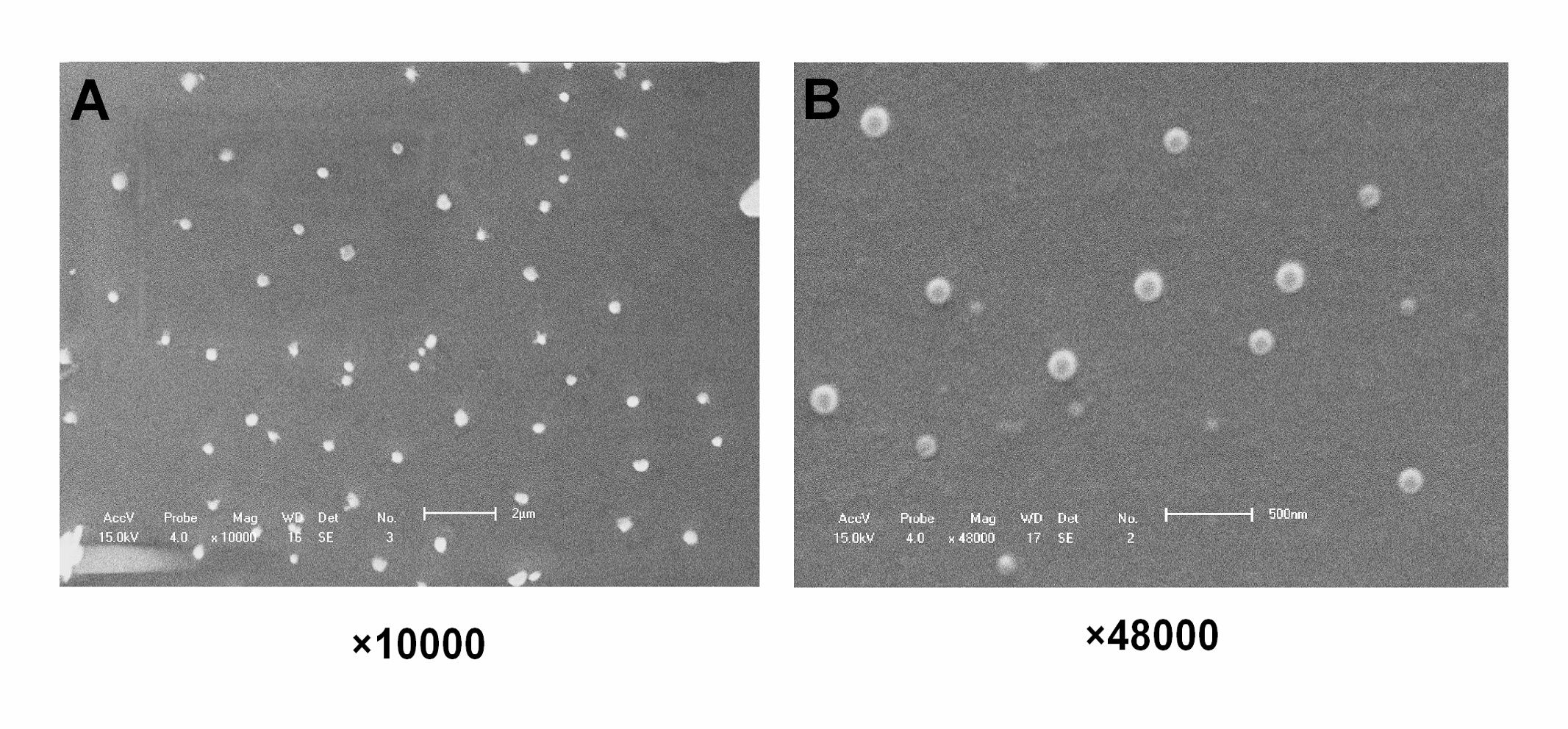


Figure S1 NPs spherical shape was analyzed by SEM scanning with 10000 and 48000 amplification as shown in（a）×10000 amplification（b）×48000 amplification

**Fig.S2 5-FU NPs size analysis**


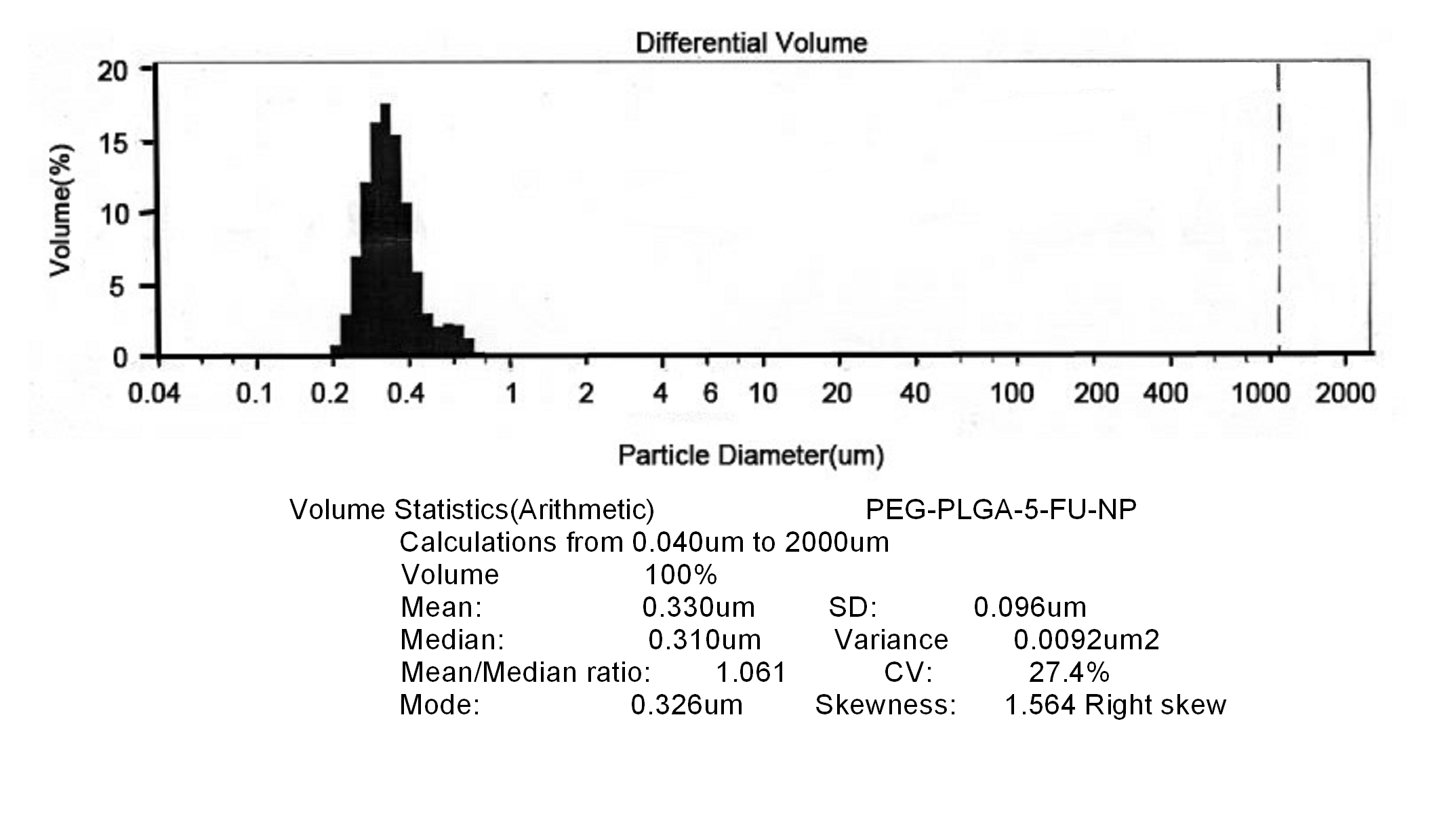


Figure S2 As shown in the results of LS particle size analyzer, the average particle size of drug carrying microsphere obtained by means of high shearing emulsification reaches 310nm. Furthermore, as examined from the particle size distribution, the length of 70 % nanoparticles is less than 385nm with the distribution range of 255~469nm.

**Fig.S3 Cell cycle blocked by 5-FU-NPs**


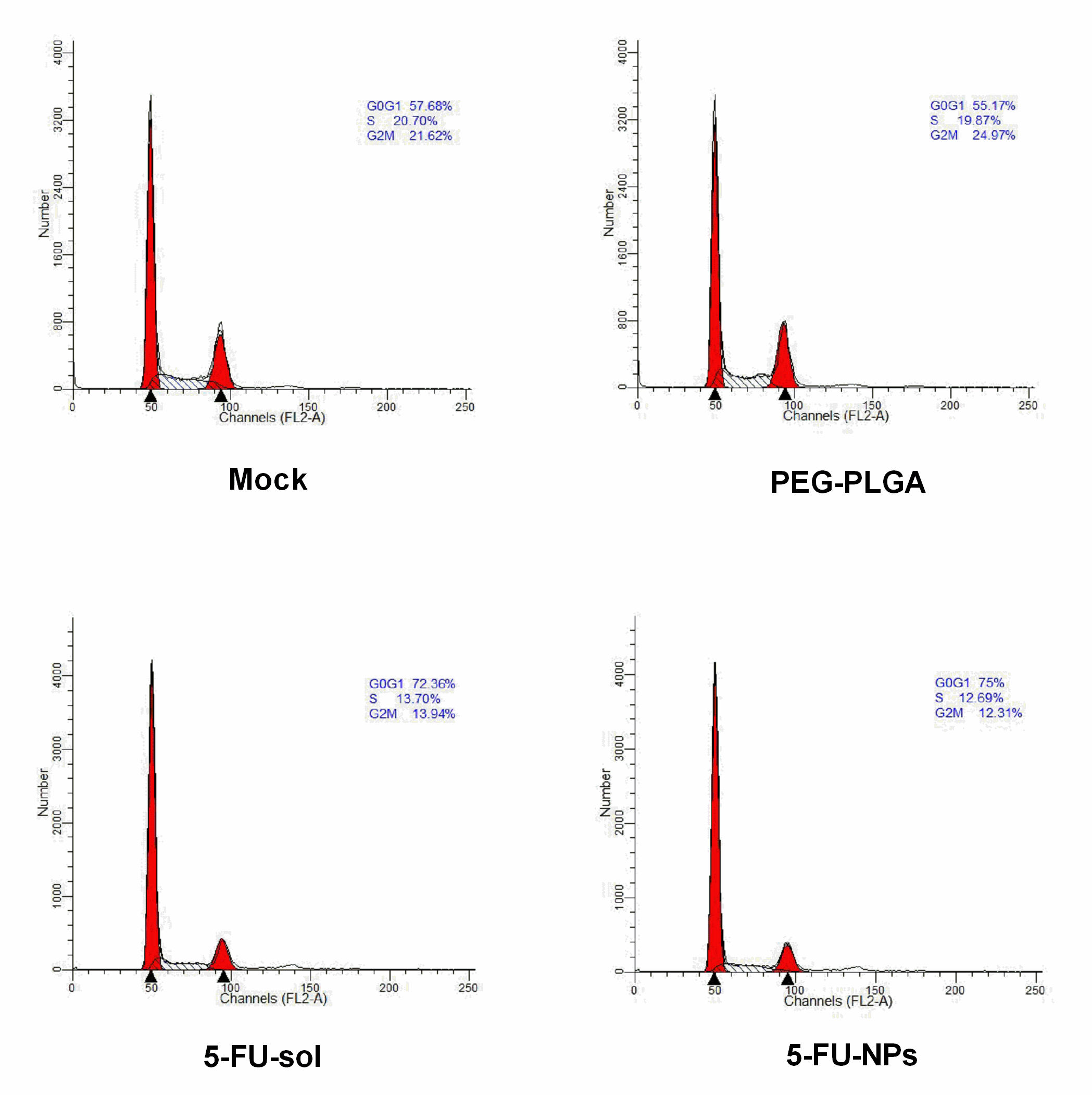


Figure S3 HCT116 cell line were added with 5-FU-NP or 5-FU-sol or PEG-PLGA or mock and subjected to a cell cycle analysis with flow cytometry. Cell cycle were blocked partly by both 5-FU solution and 5-FU-NPs compared with control group. 5-FU-NPs enhanced the cell cycle blocking ability compared with 5-FU-sol partly, but not significantly. (a)Mock (b)PEG-PLGA (c)5-FU-sol (d) 5-FU-NPs

**Fig.S4 Apoptosis promoted by 5-FU-NPs**


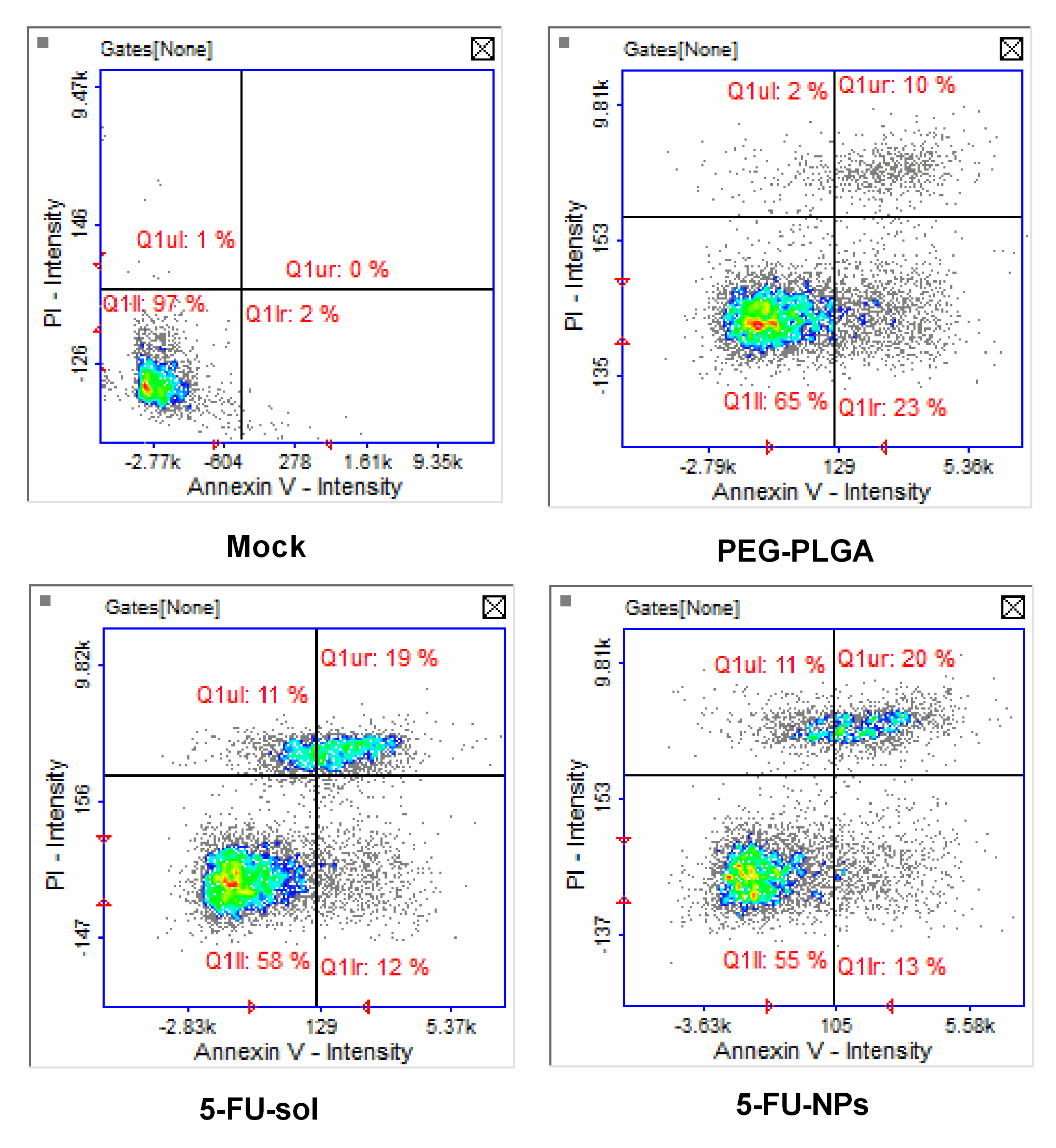


Figure S4 HCT116 cell line were added with 5-FU-NP or 5-FU-sol or PEG-PLGA or mock and subjected to a apoptosis analysis with flow cytometry. The results suggested that 5-FU-sol and 5-FU-NPs might promote apoptosis of colon cancer cells compare with mock or PEG-PLGA. 5-FU-NPs might enhance the promotion of apoptosis ability of 5-FU compared with 5-FU-sol, but not significantly. (a)Mock (b)PEG-PLGA (c)5-FU-sol (d) 5-FU-NPs

**2. Supplementary methods**

**Laser size Analysis of Particle**

Particle size distribution is determined by laser size analysator (LS-13320 laser size analysator, BeckmanCoulter, USA). Each nanoparticle preparation was analyzed in duplicate with 30 readings per nanoparticle sample suspended in distilled water.

**Flow cytometry and cell cycle** **analysis**

Single Cells were prepared for analysis of cell surface marker expression by digesting

with pancreatin before analysis. Cells were then detached from plates by incubation

with enzyme-free cell dissociation buffer (Invitrogen). Cells were washed with cold

10 mmol/L PBS and resuspended in 1× binding buffer (BD Biosciences, SanJose, CA)

at a concentration of 1 × 10 6 cells/mL. cells were subjected to direct immuno fluorescence staining (APC-conjugated anti-CD133, PE-conjugated anti-CD44), followed by fl ow cytometric analyses. The samples were analyzed using a NucleoCounter NC-3000 analyses (ChemoMetec, Denmark). The experiments were repeated at least three times.

**Flow Cytometry** **apoptosis analysis with PI and annexin V staining**

Harvest the cells after added with mock, PEG-PLGA, 5-FU–sol and 5-FUNPs, the incubation period and wash in cold PBS. Re-centrifuge the washed cells, discard the supernatant and resuspend the cells in 1X annexin-binding buffer. Determine the cell density and dilute in 1X annexin-binding buffer to ~1 × 10^6^ cells/mL, preparing a sufficient volume to have 100 μL per assay. Add 5 μL Alexa Fluor*488 annexin V and 1 μL 100 μg/mL PI working solution to each 100 μL of cell suspension. Incubate the cells at room temperature for 15 minutes. After the incubation period, add 400 μL 1X annexin-binding buffer, mix gently and keep the samples on ice. As soon as possible, analyze the stained cells by flow cytometry.
